# Understanding the Effects of Gut Microbiota Dysbiosis on Nonalcoholic Fatty Liver Disease and the Possible Probiotics Role: Recent Updates

**DOI:** 10.7150/ijbs.56214

**Published:** 2021-02-08

**Authors:** Ashiq Khan, Zitong Ding, Muhammad Ishaq, Ali Sher Bacha, Israr Khan, Anum Hanif, Wenyuan Li, Xusheng Guo

**Affiliations:** 1 School of Life Sciences, Probiotics and Biological Feed Research Centre, Lanzhou University, Lanzhou 730000, PR China.; 2 Department of Microbiology, Balochistan University of Information Technology Engineering & Management Sciences Quetta 87300, Pakistan.; 3 School of Life Sciences, Institute of Microbiology Lanzhou University, Lanzhou 730000, PR China.

**Keywords:** Nonalcoholic fatty liver disease, nonalcoholic steatohepatitis, gut microbiota, probiotics, dysbiosis, small intestinal bacterial overgrowth.

## Abstract

Nonalcoholic fatty liver disease (NAFLD) is leading chronic liver syndrome worldwide. Gut microbiota dysbiosis significantly contributes to the pathogenesis and severity of NAFLD. However, its role is complex and even unclear. Treatment of NAFLD through chemotherapeutic agents have been questioned because of their side effects on health. In this review, we highlighted and discussed the current understanding on the importance of gut microbiota, its dysbiosis and its effects on the gut-liver axis and gut mucosa. Further, we discussed key mechanisms involved in gut dysbiosis to provide an outline of its role in progression to NAFLD and liver cirrhosis. In addition, we also explored the potential role of probiotics as a treatment approach for the prevention and treatment of NAFLD. Based on the latest findings, it is evident that microbiota targeted interventions mostly the use of probiotics have shown promising effects and can possibly alleviate the gut microbiota dysbiosis, regulate the metabolic pathways which in turn inhibit the progression of NAFLD through the gut-liver axis. However, very limited studies in humans are available on this issue and suggest further research work to identify a specific core microbiome association with NAFLD and to discover its mechanism of pathogenesis, which will help to enhance the therapeutic potential of probiotics to NAFLD.

## Introduction

Nonalcoholic fatty liver disease (NAFLD), the most prevalent liver disease, which is portrayed by the deposition of fats in the hepatocytes that ranges from simple hepatosteatosis to nonalcoholic steatohepatitis, fibrosis, cirrhosis, as well as hepatocellular carcinoma 1, 2. Currently, NAFLD is becoming a global health concern which accounts for 25% prevalence worldwide and more than 30% prevalence is projected among the adults by 2030, due to the dominance of obesity, unhealthy dietary patterns and sedentary ways of lifestyle 3-5. Its incidence rate has increased with non-communicable infections, including cardiovascular disease, type 2 diabetes, as well as complex liver infections such as hepatic cirrhosis 6,7.

Historically, 'two-hit theory' was proposed to explain the pathogenesis of NAFLD, which revealed that oxidative stress is the triggering factor of hepatic injuries which develops from the simple steatosis to nonalcoholic steatohepatitis (NASH) followed by severe liver damage and cirrhosis 8,9. However, studies in the recent decades have highlighted multiple risk factors, including gut microbiota dysbiosis, genetic, unhealthy diet, metabolic, insulin resistance (IR), environmental, host-derived features like age, ethnicity, gender, antibiotic use, alcohol consumption, oxidative stress, inflammatory states, lifestyle and so on. All these factors significantly contribute to NAFLD and hepatic steatosis and several molecular pathways have been identified in the progression of NAFLD 10-14. Among these abnormal conditions, alteration of gut microbial populations has been recognized as the most common risk factor for NAFLD, obesity, and diabetes 15-17. Further, NAFLD patients have found with higher dominance of small intestinal bacterial overgrowth in the gut 18, and this leads to gut dysbiosis 19.

Recent findings have shown that dysbiosis of gut microbiota also disturbs the hepatic carbohydrate, lipid metabolism and affects the balance among the pro and anti-inflammatory effectors in the liver, thus prompting NAFLD and its development to NASH 2. However, the comprehensive mechanism of this severe liver disease is so far mysterious. Numerous new features remain yet to be revealed particularly to the specific functions of the gut microbiota 20,21. Resolving these issues would be a milestone for the treatment of NAFLD and other gut microbiota related syndromes. Therefore, it is necessary to completely recognize the pathogenesis of this disease and the role of gut microbiota in its progression, which would possibly paw the way to discover new therapeutic strategies for control of NAFLD 2.

There is a growing interest in healthy gut microbiota and the restoration of dysbiosis gut microbiota. The probiotics are commonly used as an appealing approach due to their long traditional use, health-promoting properties and enormous literature supports. Evidence suggests that probiotics could regulate intestinal microbiota and can be preferred as a novel preventing or treatment approach for NAFLD and other chronic liver diseases 22,23. Several latest findings have shown that probiotics have the tendency to change the dysbiosis gut microbiota to the normal and could be a potential treatment approach for the management of NAFLD 24-26. As there is a close anatomical and functional connection among the gut and liver as well as microbial dysbiosis ensues NAFLD and probiotics could modulate this dysbiosis. Therefore, in this review we discussed the updated literature, significance of gut microbiota, its modulation and major proposed mechanisms of pathogenesis in dysbiosis gut and its progression to NAFLD. In addition, we also reviewed the role of most commonly used probiotics for the management of NAFLD.

## How Gut Microbiota Modulations Links in Progression of NAFLD?

Gut microbiota is a complex ecosystem of microbes that lives throughout the gastrointestinal tract. It is the main site of microbial colonization and a diverse biome which consists of approximately 2,000 distinct bacterial species and collectively has a genome of about 150 fold more genes than in the human genome. Majority of the human gastrointestinal bacteria are composed of phyla *Bacteroidetes, Firmicutes, Proteobacteria, Fusobacteria, Actinobacteria* and *Verrucomicrobia* and *Cyanobacteria* 27. Generally, *Firmicutes*, *Actinobacteria, Bacteroidetes, Ruminococcaceae, Verrucomicrobia* and *Proteobacteria* are more dominated in the gut 28,29, and in a study authors found that healthy individual fecal accounts for about 80-90% of bacterial community 29. Altogether these microbes have been developed along with the human 30.

Research has explored the composition of gut microbiota in patients with NAFLD relative to healthy individuals (**Table 1**), and identified the trends that can be associated with the development of NAFLD 10,14. This shows that gut microbiota composition is adversely affected in patients with NAFLD. Gut microbiota in healthy individuals mostly has a balance among the microbiota, which perform vital functions, such as immune system regulation and detoxification. However, in NAFLD and other gut diseases, this balance among the major phyla disrupts (dysbiosis). Many of these bacterial groups of gut microbiota have been shown to either increase or decrease in NAFLD and produced various toxic metabolites, inflammation, oxidative stress and ultimately leads to liver cirrhosis.

In recent years, advances in technology have shown that human gut microbiota is extremely variable in abundance and composition and play significant role in supporting the human health and more varying in perspective of altered conditions (i.e. dietary, environmental and immune) which can lead to development of many diseases like obesity, NAFLD and hepatocellular carcinoma 4, 31-33.

Relatively higher abundance of genera, *Fusobacteria*, lower abundance of *Oscillospira* and *Ruminococcus* of *Ruminococcaceae* and *Coprococcus* of *Lachnospiraceae,* have been shown in NAFLD patients 34. Further bacterial species were identified in NAFLD patients, including *Proteobacteria, Escherichia* and *Enterobacteria* 11, also *Bacteroides* were more in NASH patients paralleled to healthy individuals 19. Similarly, authors in a study have shown a lower diversity of microbiota in feces of children infected with NAFLD and an increased number of *Prevotellacopri* and a lowest α- bacterial diversity was linked with severe liver fibrosis parallel to control 16.

In addition, latest published study identified a decrease in alpha-microbial diversity and beta microbial diversity in a moderate stage of nonalcoholic fatty liver disease. Interestingly, they observed a decline in alpha (α) and the rise in beta (β) microbial diversity in the severe stage of this disease (NAFLD-cirrhosis) 35. Likewise, a recent study showed a higher proportion of *Bacteroides* as compared to the *Lactobacillus* as well as *Bifidobacteria* in the gut of the liver fibrosis patients 36. These evidences shows that gut microbiota modulation may have a significant role in the development of NAFLD. Continuous advances in sequencing tools, as well as phonemic research now make this feasible to give a more comprehensive detailed functional dimensions of gut microbiota and its effects on the host liver phenotype 35. Thereby, alterations of the microbial population in the gastrointestinal tract undoubtedly have potential effects on pathophysiology of gut diseases and studies are essential to discover innovative probiotics therapies that can restore the normal gut microbiota.

## Gut-liver axis Malfunction and its Impacts on NAFLD?

The gut-liver axis is a close anatomical as well as the functional connection of the gastrointestinal tract (GIT) and liver. This axis is explained to increase the connections among the metabolites of the gut microbiome and liver surface receptors, which can activate a cascade of the severe events, leading to insulin resistance (IR), liver inflammation and enhanced liver fibrosis 47. There is unique co-ordination among the gut and liver. Disruption of this axis has shown to play an essential role in the pathogenesis of several liver infections including NAFLD. The disruption includes gut barrier disturbance, bacterial translocation (BT) and inflammatory reactions in the liver, for example, Toll-like receptor signaling, activation of inflammasome as well as alterations in the composition of bacterial metabolites 2,48,49. However, it is needed to clarify that this association is whether causal and further to elucidate the mechanism through which gut dysbiosis plays a role in the pathogenesis of NAFLD 50. The altered gut microbes produce ammonia, ethanol and acetaldehyde and these toxic products may affect the liver functions via endotoxin release from Gram-negative bacteria or liver metabolism 48.

Cross talk among this axis is progressively recognized and reinforced via the correspondent increase in the incidence of immune disorders, gastrointestinal and liver diseases 51. It is explored that gut microbiota dysbiosis and impairment of gut mucosal barrier affect the cross-talk between the gut and liver via small intestine bacterial outgrowth, which causes intestinal inflammation and could result in NAFLD 39. It is also proposed that an obese individual gut microbiome can increase the pathogenesis of NAFLD by affecting lipid metabolism as well as enlarged its capacity to harvest more energy from the diet 9,17. Therefore, due to these fluctuating features, modification of gut microbiota has produced a huge potential interest in the use of novel probiotics specifically for the prevention and treatment of chronic NAFLD 48. Advances in technologies as well as understanding the research area of the gut-liver axis could boost research into gut microbiome based therapeutic approach to improve the management of this disease 52. Consequently, studies are essential to address the link between the gut-liver axis and NAFLD and to elevate the role of potential probiotics and its mechanism of action on the gut liver axis for governing of NAFLD.

## The Links between Small Intestinal Bacterial Overgrowth and Leaky Gut and NAFLD Pathogenesis

Small intestinal bacterial overgrowth (SIBO) is defined as an imbalance of normal gut microbiota in the small intestine 53. This causes the disruption of gut microbiome diversity, its composition and associated with progression to NAFLD and other intestinal as well as extra intestinal diseases 13,17,50. Among many potential contributions of gut microbes to liver diseases, small intestinal bacterial overgrowth (SIBO) has been comprehensively studied. Malfunction of gut microbiota can cause intense systematic infections 54. Especially, dysbiotic microbes are often identified among the obese individuals and considered as a key risk factor for the development of NAFLD 17,24. In dysbiosis and leaky gut, bacterial derived metabolic products (e.g. lipopolysaccharide) could induce inflammation of adipose tissues, hepatic steatosis, as well as hepatic inflammation 55,56.

Gut dysbiosis is associated with the pathogenesis of NAFLD and its severity is connected with a larger abundance of inflammatory genes encoding bacterial products. Variations in the intestinal microbiome can contribute to the pathogenesis of NAFLD and can be used as biomarkers of disease and its severity 16. Both NAFLD and obesity are associated with a larger concentration of Gram-negative bacterial species in the gut 11. Gut leakiness is commonly caused by dysfunction of the intestinal barrier structures, which prompted intestinal inflammation as well as TNFR-I (TNF receptor-1) signaling in epithelial cells and it is showed that because of increased gut permeability, endotoxin, metabolic products, translocate into the liver 57, and activate the immune cells (Kupffer cells) via the receptors (TLR4 or TLR9). Then these cells produce various inflammatory cytokines and worsen the disease 58. The role of other TLRs in the regulation of NAFLD remains mostly unknown, so it needs to be studied and further better understanding of tool- like receptors (TLR) signaling pathways in liver, which will help to explain the mechanisms of the liver tumorigenesis and will propose novel therapeutic targets for hepatocellular carcinoma 2.

Animal model investigations have evaluated the significance of gut dysbiosis in the development of NAFLD. Studies also determined that translocation of dysbiotic microbiota deteriorated the hepatic steatosis and therefore increased the progression of NAFLD 11, 37. A recent study has shown that gut microbiota from the obese human donors induced hepatic steatosis in germ-free mice via influencing lipid metabolism as well as transcriptional profile 59. Authors in a study determined, that germ-free mice fed with high-fat diet were inoculated with gut microbes of NASH patients, showed a worsened NASH phenotype, as indicated by increased hepatic steatosis as well as inflammation 60. In dysbiosis, the host is compromised and microbiota is unable to control the normal homeostasis and increase the intestinal permeability. These changes expose the liver to various exogenous and endogenous antigens through the gut-liver axis 61. It is shown that SIBO might increase the absorption of endotoxins which initiate the pro-inflammatory as well as pro-fibrogenic effects on the liver 62. Moreover, unusual changes like increased peroxidation of lipid, as well as oxidative stress, are effective in the raise of bacterial translocation (BT) 63. Evidence also showed that compromised antimicrobial defense mechanisms could increase BT, results in portal hypertension and liver cirrhosis 64.

**Figure 1:**
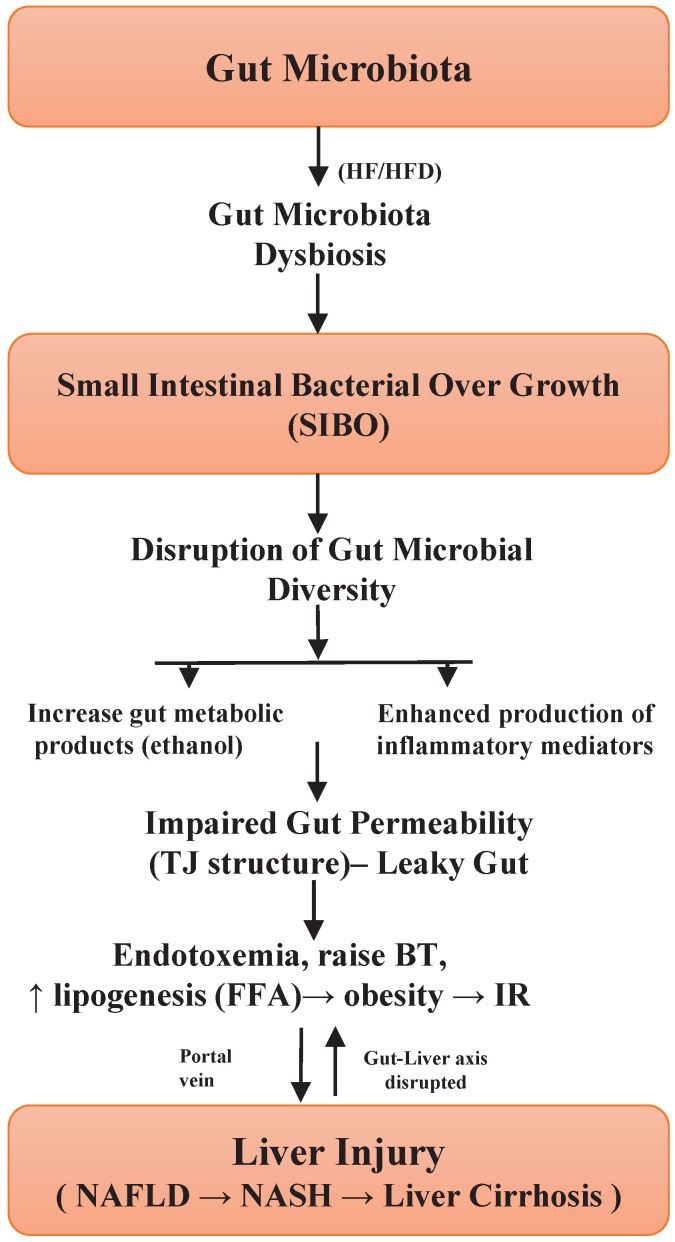
Illustration of how small intestinal bacterial overgrowth impacts the gut microbiota dysbiosis and progression of NAFLD.

Tight junction's proteins are mucosal barriers that inhibit the paracellular translocation of bacteria. Impairment in the integrity of this protein leads to an increase in the translocation of microbial metabolic products, for example, lipopolysaccharide (LPS) in the bloodstream and ensuing endotoxemia, can persuade inflated intestinal permeability and subsequently liver inflammation 39,65. In addition, alterations in the intestinal tight junction structure have been studied in liver cirrhosis patients, however, the exact pathophysiology and dysfunction of the gut barrier are unclear. The maintenance of tight junction integrity would be a good approach to prevent or treat NAFLD 66, and other gut diseases. A study conducted in humans showed a significant relationship between gut permeability, SIBO, systemic inflammation and NAFLD 67, hence suggesting the importance of increased gut permeability. The severity of NAFLD is thus linked with SIBO and dysbiosis of gut microbiota. Based on these encouraging outcomes, studies are necessary to establish an exact cause and effective association between gut dysbiosis and NAFLD 2. These results give credibility to hypothesize that improvement of the gut barrier disruption could help to alleviate the progression of the disease. **Figure 1**, highlights the significant role of SIBO in gut microbiota dysbiosis as well as the development of NAFLD 13,17,25,35,55,61. Briefly, unique probiotic treatment approaches should be implemented, which can regulate the small intestinal bacterial overgrowth in the gut and thus could reduce the gut dysbiosis and this will ultimately mitigate the disease.

## Mechanisms in Dysbiotic Gut Microbiota: Impact on NAFLD?

Dysbiotic gut microbiota and its several metabolites and endotoxins might be the characteristic of NAFLD progression through numerous mechanisms. Following are some major mechanisms showing a significant role in the progression and development of NAFLD.

## Crosstalk between Dysbiotic gut Microbiota and its Metabolites

### Bile acids (BAs)

Bile acids are amphipathic in nature, produced in the liver from cholesterol 68. These are secreted in the biliary tract and reach the small intestine via the duodenum, combine with other components of the biliary tract, enable the emulsification, digestion, absorption of dietetic fats, cholesterol as well as emulsify fat-soluble vitamins 52. Bile acids (BAs) are known as a significant regulator of lipid metabolism, glucose and energy homeostasis. Regulatory properties of bile acids have been well studied concerning the farnesoid X receptor (FXR), identified as a transcription factor (NR1H4) and Takeda G protein-coupled membrane receptor 5 (TGR5). These bile acid receptors regulate its endogenous synthesis and release as well as regulate several host metabolic pathways. It controls more metabolic functions via alterations in its transcriptional gene expression 69, 70. It is showed that almost 95% of bile acids keenly reabsorb in the last part of ileum then transfer back to the liver 71. While residual 5% BAs deconjugated, dehydroxylated and dehydrogenated by colonic microbiota to procedure secondary bile acids (deoxycholic acid, lithocholic acid and ursodeoxycholic acid), reached the liver then to portal circulation through passive absorption 70,72.

**Figure 2:**
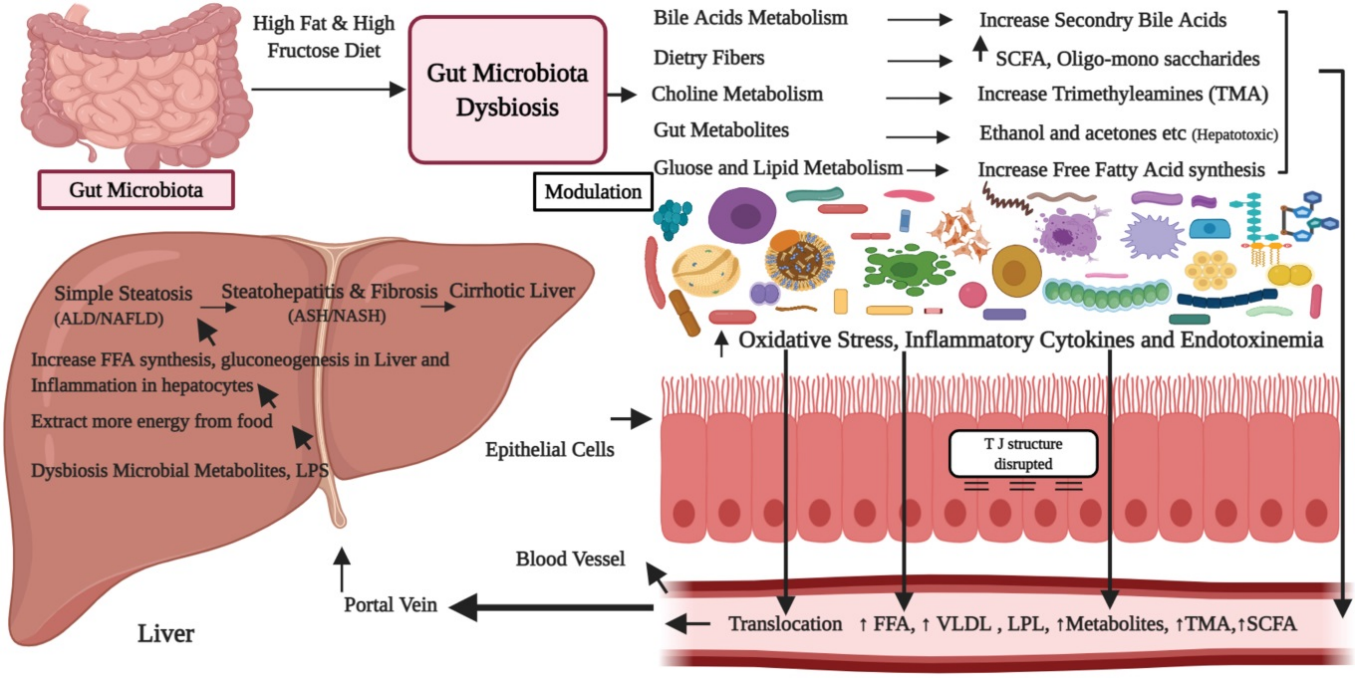
Proposed mechanisms showing the role of high-fat and sugar diets in the progression of gut microbiota dysbiosis and development of the nonalcoholic fatty liver disease.

The deconjugated BAs are less effective in formation of micelle and emulsification of ingested lipids parallel to conjugated BAs, so reduce its proficiency for fats absorption 73. The connection between gut microbiota and NAFLD, which is moreover dependent on altered BAs metabolism via the gut microbiota 74. Besides, gut microbiota could affect the homeostasis of BAs pool through deconjugating, also metabolizing primary BAs to secondary bile acids in gut, these toxic molecules take part in the modulation of lipids as well as energy metabolic pathways (**Figure 2**), and altered bile acid signaling receptors (FXR/TGR5), that can cause NAFLD 75-77. However, little is known, which gut microbe is involved in this conversion. In addition, studies indicated that bile acids and gut microbiota are closely related and maintain each other. BAs directly regulates microbiota via binding to the FXR receptor, prompt the antimicrobial peptides (angiogenin1) and peptides of (RNase family 4), which directly inhibit the overgrowth of gut microbiota and subsequently mucosal barrier dysfunction 78.

Gut microbiota dysbiosis alters the balance among primary bile acids as well as secondary bile acids and their successive enterohepatic cycling, which metabolic properties are not precisely clear. An imbalance in bile acids and intestinal microbiota cause a cascade of immune reactions, inflammation and development of liver diseases 52. Authors in a study have shown that NASH patients have altered bile acids composition with a preponderance of secondary bile acids 76, 79. It is also revealed that the altered BAs destruct the cellular membranes via interaction with the membrane phospholipids, which results in bactericidal action 8. Definitely, BAs are specific molecules for a G-protein-coupled receptor, similarly activates FXR (NR1H4), which downstream targets and shows a key role in regulation of hepatic *de novo* lipogenesis, plasma triglyceride turnover also very-low-density lipoprotein-triglyceride distribution 80. TGR5 hinder secondary bile acids, stimulates glucose homeostasis via inducing the secretion of glucagon-like peptide 1 69. In a study supplementation of definite TGR5 agonists reduces triglyceride profile in serum as well as in the liver, thereby decreasing liver steatosis 81. Maintaining the bile acid metabolism and microbiota can therefore, halt the progression of NAFLD 82. Moreover, an association between gut microbiota and bile acids offers an important indication for the gut microbiota targeted treatment for NAFLD 76,83. Thus, novel bile acids (BAs) regulatory probiotic strains are essential which may ameliorate the gut microbiota by regulating BAs pool and FXR activation.

### Choline

Choline is an essential phospholipid and part of the cell membrane, shows a key role in lipid metabolism in the liver, in the assembly of very-low-lipoprotein. It encourages the transport of lipid from the liver 84. Choline halts abnormal lipids accumulation in the liver, whereas deficiency of choline produces abnormal phospholipid, defective very low-density lipoproteins and causes alteration in the bile acids circulation (**Figure 2**), which commonly leads to hepatic steatosis. Several other factors also affect the bioavailability of choline, including food intake, estrogen status, single-nucleotide polymorphisms (SNPs) variation in genes for the *de novo* choline metabolism 85. Nevertheless, gut microbiota metabolized the dietetic choline into a variety of toxic metabolic products, for example, trimethylamine and therefore can decrease the choline bioavailability 86.

Gut microbiota produce enzymes (e.g. choline-TMA lyase, glycine betaine reductase), which catalyze the dietetic choline in toxic methylamines (dimethylamine and trimethylamine). Liver uptake these amines and convert into trimethylamine-N-oxide, could produce liver inflammation as well as liver damage 87,88. Evidence showed that choline-deficient food stimulates liver steatosis, reversible through choline infusion 89. Hence, gut microbiota dysbiosis could stimulate NASH by reducing dietary choline levels, as well as raising toxic methylamines 85. Studies described that feeding mice deficient with choline for 4 weeks induct NASH like disorder and produced many clinical signs and serum markers, high concentration of liver enzymes, hepatic triglycerides, lipid peroxidation as well as weight loss in mice 52, 90. However, experiments conducted in germ-free mice indicate that mice do not produce such toxic methylamines, which support the important role of intestinal microbiota in the transformation of dietary choline to its intermediate compounds 91.

Normally, choline is converted into the phosphatidylcholine, for example, a lecithin compound in the host, which causes excretion of low-density lipoproteins particles from the liver and prevents hepatic triglycerides accumulation in the liver (steatosis) 92. However, choline is also transformed to trimethylamine by gut microbiota, where trimethylamine can transfer to the liver and further change to trimethylamine N-oxide 93. Circulation of trimethylamine is implicated with a low concentration of host-derived phosphatidylcholine; an imbalance that commonly causes gut dysbiosis. Increased trimethylamine concentration has been related to liver injury due to a higher accumulation of triglycerides in the liver 94. Increased triglycerides (hepatic steatosis) results in NAFLD in humans as well as in experimental animal models 95. Further, NAFLD patients are associated with SIBO and its demand for phosphatidylcholine increases, for its growth as well as division; this might contribute to the deficiency of choline in the host and subsequently contribute to NAFLD as well as NASH 2. Accordingly, a close and benefited relationship exists among gut microbiota and choline metabolism, which provides a significant role of gut microbiota-targeted treatment for NAFLD 83. Thus, modifying the metabolic profiles of gut microbiota may serve as unique microbiome-based strategies for the management of NAFLD 96.

### Ethanol and acetaldehyde

Gut microbiota produces several potential hepatotoxic molecules, for example, ethanol, acetaldehyde, ammonia and phenols, which are transported to the liver via portal circulation system. The gut mucosa directly absorbs maximum ethanol from food, beverages, stomach as well as small intestine via simple diffusion, while the biggest part of the alcohol (ethanol) produced by microbial fermentation comes from large intestine through the systemic circulation 11. Interestingly, studies showed that NAFLD is progressed by increased circulating as well as luminal levels of ethanol, its metabolites, like acetaldehyde and acetate 11,96. The gut microbiota plays an important role in pathogenesis by numbers of alcohol-producing bacteria largely *Escherichia coli*, which has been established to raise gut epithelial permeability. In addition, the elevated level of serum ethanol (**Figure 2**), play a major role in oxidative stress, liver inflammation and development of NASH 39. Besides *E. coli,* other bacterial genera, like *Bacteroides*, *Bifidobacterium*, as well as *Clostridium*, are also shown to produce alcohol, causes important ethanol mediated injury 11. Ethanol is most common as well as frequent metabolite of various hetero-lactic organisms and endogenously gut microbes produced it. It is responsible for physiological and morphological differences, especially causes activation of macrophages, pro-inflammatory cytokines production, through which gut bacteria might interrupt the intestinal barrier, leads to small intestinal bacterial overgrowth and therefore enhances translocation of endotoxins into the portal circulation 97. Further, apart from the disruption of the gut barrier integrity, it is also associated with the secretion of pro-inflammatory liver cytokines and pathogenesis of NAFLD 98. However, the precise mechanisms of the present hypothesis need more study 28.

Likewise, acetaldehyde and its metabolites also stimulate the Kupffer cells, which activate the innate immune system, increase the production of nitric oxide, cytokines, which causes the production of ROS 99. Acetaldehyde is involved in weakening of the gut tight junction proteins, compromises gut barrier integrity and allowing translocation of the microbial products 100. It is also linked with down-regulation of the expression of AMPs in the gut 101, causes its permeability, which leads to disruption of gut barrier functions, proliferates the oxidative stress and ultimately induces the liver injury 102. The liver reacts to circulating ethanol by up-regulating its ethanol metabolic pathway 103. Gut microbiota as well as enterocytes produce alcohol metabolizing enzymes (alcohol dehydrogenase), that co-metabolizes alcohol (ethanol) into major metabolites like acetaldehyde as well as acetate 104. This shows that modulation of gut microbiota produces various altered metabolites, which ultimately leads to liver inflammation and the onset of NAFLD.

### Short-chain fatty acids

Short-chain fatty acids (SCFAs), like acetic acids, propionic acids and butyric acids are commonly produced by fermentation of gut microbes 105. The gut microbes supply substrate for fermentation of the complex indigestible dietary polysaccharides to make SCFAs and support the host in getting maximal energy 39. These SCFAs are rapidly absorbed in the colon and have many functions in maintaining the inflammation, gut motility, glucose, lipid homeostasis and energy harvesting 106. However, overproduction of the SCFAs might promote lipogenesis in the liver as they use it as a substrate for lipogenesis 107.

The various gut metabolites produce mutable effects on the host and some of them could be beneficial for synthesis of signaling molecules like butyrate 39, that not only supply energy for gut mucosal cells but also prevent the production of cytokines and reduce the incidence of chronic metabolic inflammation via GPR43 pathway 108. Qin et al*.* have revealed that the composition of gut microbiota in the cirrhosis patients is characterized by the lower number of butyrate synthesizing bacteria as compared to healthy subjects, which have anti-inflammatory characteristics 109. Butyric acid activates the peptide AMP-activated protein kinase, in the liver 110, therefore, improve the gut barrier dysfunction 111. Similarly, in a study it was shown that butyrate could efficiently improve the lipid accumulation as well as liver inflammation in animal models of NAFLD via modulation of gut microbiota and barrier functions 112, reduce the induction of induced nitric oxide synthase 113, and suppress the inflammatory pathways 114.

In the gut dysbiosis state, bacterial species of *Bacteroidetes* are identified as the main contributor to acetate and propionate production, whereas *Firmicutes* as a key producer of butyrate. Increased level of propionic acid is shown to promote gluconeogenesis in the liver 115. SCFAs triggered the expression of G protein-coupled receptors (GPRs), especially the GPR41 and GPR43, which could encourage the secretion of the peptides GLP1 116, and stimulate the genes in the liver cells that regulate the fatty acid β-oxidation pathway and also insulin sensitivity 117, thus promoting the occurrence as well as progression of NAFLD (**Figure 2**). Experiments have determined that feces of obese individuals have high levels of SCFAs then lean individuals, suggesting that gut microbiota from the obese individuals exhibit the increased capability to extract and store energy from food as compared to lean ones 118. The role of other short-chain fatty acids, for example, acetic acid as well as propionic acid, could directly infect the hepatocytes, adipose tissue, as well as central nervous system 119, which needs further study. Studies are required to validate how SCFAs can play a role in the pathogenesis of nonalcoholic fatty liver disease/NASH subjects 2. **Figure 2**, highlights the proposed mechanisms showing the role of high-fat and sugar diets in the progression of gut microbiota dysbiosis and development of the nonalcoholic fatty liver disease 2, 51, 52, 70, 92, 97, 107. In fact, high-fat diets and high sugar results in gut dysbiosis and small intestinal bacterial overgrowth (SIBO). Alterations in gut microbiota (dysbiosis) increase energy extraction from the gut and alter the normal dietetic choline, BAs, lipids and glucose metabolism. This consequently increases the production of endotoxins, pro-inflammatory cytokines, increase the gut epithelial permeability and toxins affect the gut liver axis, which finally results in NAFLD/NASH. In summary, more studies are essential to determine the significant characteristics of SCFAs and especially its role in the NAFLD and it is essential to completely understand the thorough molecular mechanisms and consequences of these hepatotoxic compounds on the liver.

## How Probiotics Role as Therapeutic Strategy for Management of NAFLD?

The gut microbiota plays a vital role in humans through supporting the gut homeostasis, protection against pathogens, enhancing the development of the immune system, also supporting in production of energy as well as micro-nutrients 120. It could be easily presumed that gut microbiota shows a fundamental role in maintaining human health 106. Indeed, changes in the composition of microbiota have been linked to several metabolic syndromes including NAFLD 10,121,122. It is hypothesized that gut microbiome restoration might be a better method for treatment of NAFLD 123-125. Probiotics have been proposed to restore the dysbiosis gut to the healthy gut microbiome and administration of probiotics can be a treatment approach for prevention and control of chronic liver diseases because they are non-pathogenic 25,26,50, and can prevent translocation of harmful bacteria and endotoxins by inhibiting their epithelial invasion, preventing bacteria adherence to mucosal surface, decreasing inflammatory responses via the production of antimicrobial peptides and furthermore stimulating the host immunity 50. Evidence showed that probiotics boost intestinal microbiota to re-establish intestinal-mucosal cross-talk which reduces the progression of NALFD 126.

A significant number of studies revealed that probiotics normalize the gut epithelial homeostasis, i.e. promote cell persistence and gut barrier functions by preventing the translocation of LPS thus alleviate eubiosis, improve gut bacterial ecology as well as adjust the immune functions 49,127. Continuing experimental trials involving probiotics in NAFLD 128, could bring more development in this research field from the basic research towards the clinical practice. Recently encouraging outcomes of probiotics are shown in pre-clinical studies in children as well as adults with NAFLD. Nevertheless, randomized controlled investigations are limited in these populations 24,129. Therefore, large scale clinical studies are essential to understand the efficacy of probiotics against NAFLD.

## Probiotics

According to FAO/WHO, Probiotics can be defined, as “live, non-pathogenic microbes when administered in the appropriate quantities, confer a benefit to the host health” 22. Probiotics are traditionally fermented foods and the *Lactobacilli* as well as *Bifidobacteria,* are the main bacteriological groups used as probiotics 130, 131. Conversely, for most of the commercially available probiotic strains the parameters such as quality control, including its characterization, viability, formulation and safety issues are rarely addressed 25. Generally, the candidate probiotic strains that to be used for treatment should strictly follow the proposed WHO guidelines before commercialization in the market 132.

The ideal candidate probiotic strain (n) must have the features to take part in indigenous colonization 133, originated from the human, non-pathogenic 134. It should hinder the growth of various pathogens, survive in the human optimum body temperature, immune to antibiotic therapy. It lives throughout the gastrointestinal tract, oppose to bile salts (pancreatic juice) 132, 133. Probiotic strains should live in duodenum environment, stimulate the immune system via the production of the antagonistic proteins (bacteriocins) to pathogens, produce short-chain fatty acids to improve the epithelial barrier and to increase the anti-inflammatory action and balance the gut microbiota 132. Additionally, the candidate probiotics should have a protecting role in the host against the oxidative stress, anti-cholesterol, anti-obesity, and anti-diabetes 132,135.

Studies illustrate that the probiotics become the source of modulation and functions of endogenous microbiota and disturbing its interaction with the host, also causes the competitive elimination of pathogens. Further, available fact shows that probiotics can inhibit obesity as well as NAFLD by improving fatty acid oxidation, promote the mucosal barrier function, mucosal recovery and regulate innate immune responses in pathological situations 23. Therefore, manipulating the gut microbiota can decrease the endotoxins and other hepatotoxic toxic compounds and further effective in reducing the inflammation, increase the fatty acid oxidation 134.

**Figure 3:**
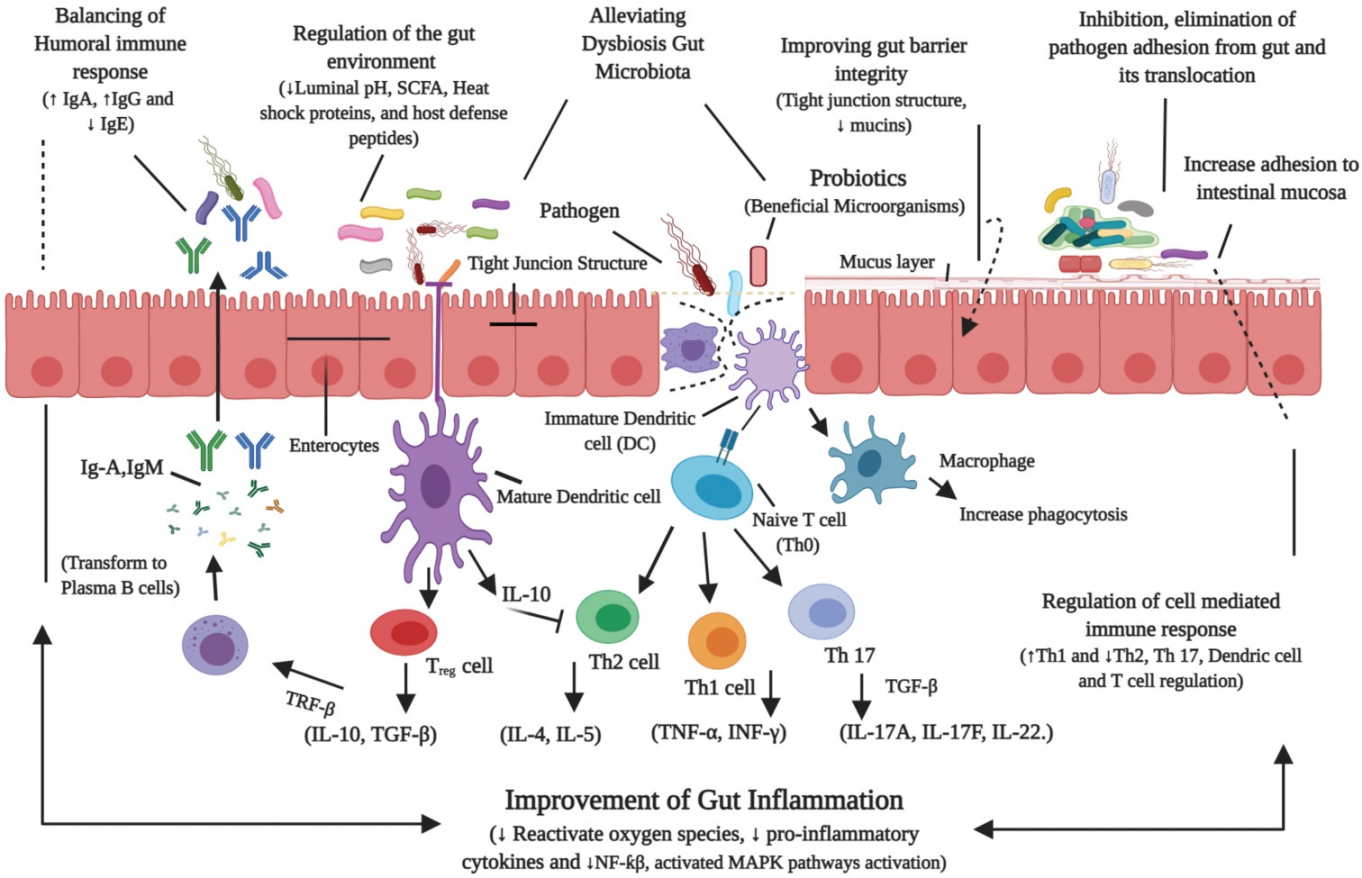
Probiotics mechanisms of action in alleviating the host gut microbiota homeostasis. Probiotics could alleviate the gut disorders (NAFLD), by modulating the gut microbiota, prevent the translocation of bacterial toxins, reduce the gut permeability and regulate the immune response.

Recently, an investigation has shown that probiotics have the tendency to alter the dysbiosis gut microbiome towards the beneficial 24. **Figure 3**, illustrates the probiotics mechanisms of action in alleviating the host gut microbiota homeostasis 23, 25, 26, 50, 120, 132, 137.

Overall, the beneficial effects of different probiotic strains on NAFLD have shown. However, it is not fully elucidated about specific probiotics, that either individually they are more effective or in combination, for the treatment of NAFLD. Therefore, future studies are needed to discover a unique and specifically effective probiotic (n) that fully attenuates or treats the key source of this disease, which will help to enhance the therapeutic potential of NAFLD.

## Therapeutic role of probiotics on NAFLD in animal model studies

Several animal model studies have shown evidence that probiotics can reduce the progression of NAFLD 134,138. They could recover the gut microbiota configuration, liver pathology via the down-regulation of serum LPS and liver TLR4. It is proposed that both alteration of gut microbiota and their endotoxemia could be involved in the development of NAFLD and probiotics can delay the development of NAFLD by restoring the gut microflora, improve the expression of tight junction proteins (occludin), which can inhibit endotoxin circulation in blood by the LPS/TLR4 signaling 139. Probiotic therapy was conducted and shown that *Lactobacillus plantarum* could alleviate the NAFLD by maintaining fat metabolism such as activating the AMPK pathway into phosphorylate ACC and further halt SREBP-1/FAS signaling, which may inhibit *de novo* lipogenesis as well as increase the fatty acid oxidation. Furthermore, treatment with* L. plantarum* NA136, Nrf-2 (transcription factor) was increased which might activate the antioxidant pathway, that could decline in pro-inflammatory cytokines and thus protect against the oxidative stress 134. Other animal model studies on NAFLD, alcoholic liver disease (ALD), along hepatic encephalopathy (HE) have described the valuable properties of certain probiotic strains on liver damage. Especially, in experimental studies of NAFLD, the importance of probiotic strains have been shown 140. It was noted that oral supplementation of *L. plantarum strains* in mice with HFD, can ameliorate the progression of NAFLD by regulating the liver functions, fecal microbiota, improves the expression and activities of antioxidant enzymes and downregulated the lipogenesis associated genes 141. Similarly, supplement with a mixture of probiotic preparation modulated the gut epithelial permeability i.e. maintain the tight-junction proteins (ZO-1 and ZO-2), attenuated inflammation and also reduced the concentration of liver triglyceride 142. Pretreatment of the individual mice with oral probiotics prevented the disruption in gut barrier function (zonula occludens-1), significantly reduced the liver damage and decreased the bacterial translocation by improving the gut mucin expression (MUC2), and inhibiting against LPS or TNF-α induced gut barrier injury 143. In a latest study, authors isolated novel probiotic strain (*Bifidobacterium animalis* subsp. *lactic* Bb12), from the healthy Mongolian youngsters and orally administration of *B. animalis* subsp. *lactic* V9, significantly mitigated the HFD-induced increases in ALT, AST, ameliorated hepatic steatosis, reduced the transcription of PPAR-α, SREBP-1c, FAS. Further, V9 administration restored the expression of hepatic phosphorylated-AMPK and suppressed the inflammatory cytokines like, IL-6, IL-1β, TNF-α in the HFD fed rats. The anti-inflammatory properties of *B. animalis* subsp.* lactic* was shown to be connected with suppression of hepatic gene expression (e.g. TLR4, TLR9 and NLRP3 and ASC mRNA), in the HFD rats. These encouraging results shows that V9 alleviate NAFLD via regulating *de novo* lipid synthesis, suppressing the inflammation by AMPK as well as TLR-NF-κB pathways 136. More excitingly, it was showed that probiotic mixture can promise to treat the pathogenesis of NAFLD and could mitigate the high fat/high sugar diet-induced obesity via increasing the gut beneficial bacteria and reducing the pro-inflammatory gut bacteria 144.

In addition, researchers identified that multi-strain probiotics can prove more valuable than single probiotic against fat accumulation and microbiota modulation in diet-induced obesity 125, 145. In a study, a mixture of *Bacillus animalis* VKB/* Bacillus animalis* VKL/*Lactobacillus casei* strains, revealed more effective than a strain* Lactobacillus casi* in reducing the weight of the obese mice, cholesterol level, modulating gut microbiota in energy rich diet and further restoring the morphology of liver 146. In another recent study, probiotics mixture was supplemented in combination, which significantly alleviated the gut microbiota, reduced the fats in liver, alleviated aminotransferase activity and improved the level of TNFα, IL-6, IL-8 in HFD-induced NAFLD rats. It also decreased the expression of HMG-CR and up regulate the PPAR-α levels whereas down regulate the PPAR-γ as well as SREBP-1C levels 147. Similarly, it has been found that a mixture of probiotics can alleviate NAFLD by improving the energy, fat metabolism and regulating the gut microbiota 148. However, further research is necessary to explore specific probiotic strains and understand its role in combination for the management of gut microbiome in NAFLD patients. **Table 2**, highlights the role of probiotic strain (n) in experimental studies to treat NAFLD.

## Therapeutic role of probiotics on NAFLD in human studies

Experimental studies on NAFLD showed the importance of probiotics. However, less data is available on humans as compared to animal studies. Preliminary data revealed considerably the effectiveness of several probiotics and compared them with control in NAFLD patients 59. In addition, two double-blind and placebo-controlled studies, shown an important decrease in liver enzymes (ALT, AST), triglycerides, LDL-C, with supplementation of probiotics in obese children 149. Malaguarnera et al*.* found that oral supplementation of *Bifidobacterium longum* along with the fructo-oligosaccharides and modification of lifestyle, improved alanine aminotransferase level (ALT), cholesterol, TNF-α, lipoprotein, endotoxins in serum profile, improve the insulin resistance (IR) and hepatic steatosis index in NASH patients 150. These encouraging results intensely indicate the benefits of probiotics in the medication of NAFLD. However, as shown in a meta-analysis, still, more studies are required about the usage of probiotics in treatment of NAFLD 151.

Study showed that the maintenance of gut flora might be a novel therapeutic approach in regulating of NAFLD and probiotics can modulate the gut microbiota 26. Further, a meta-analysis confirmed that probiotic treatment effectively reduced the liver enzyme profile, however, their effects on the improvement of lipid profile were not significant 152. Further, some studies did not show the supportive effects of probiotics and have shown conflicting clinical findings for several probiotic strains and their formulations 153, particularly in treatment of gut barrier dysfunctions, as indicated in patients with abdominal surgery. Altogether following side effects (i.e. usually noted BT, gut colonization with enteric bacteria also septic morbidity), after surgery were not recovered using an oral dose of *Lactobacillus plantarum* 299v for one week 154. The rules for studying liver diseases given by American Gastroenterological association do not encourage probiotics for the treatment of NAFLD 155. This indicates that probiotics must be safe and effective when consuming for management of the NAFLD. However, current research might give more support to it in the future. Although, too much literature is available on the subject area and undoubtedly the animal models on NAFLD and numerous probiotic strains have been studied in various studies. Though, it is tough to conclude about the accurate effect of probiotics on NAFLD patients. Therefore, detailed large-scale clinical studies are required in human volunteers with NAFLD to insure the benefits of novel probiotic strains/therapeutic approaches on dysbiosis gut microbiota and in the treatment of NAFLD. **Table 3**, highlights the role of probiotic strains in clinical studies to treat NAFLD patients.

## Conclusion and Prospects

In summary, human gut microbiota maintains normal health and gut functions. Its alteration leads to pathogenic microbes and gut-derived products, which play a significant role in the progression of diseases in humans, such as NFALD, obesity, diabetes and other metabolic syndromes. Although NFALD is a multi-factorial liver disease and gut microbiota alteration (dysbiosis) is considered a novel factor to be involved in its pathogenesis, studies have shown the mechanism of this disease, but its other causality is yet to be established. There is a need to explain the exact mechanisms of gut liver axis interactions, gut dysbiosis and feasible therapeutic options. Gut microbiota restoration will be a milestone in the management of this disease. A better understanding of the individual gut microbiome, disease pathogenesis and the ideal probiotic strain(n) would be the best approach for the management of NAFLD. Encouraging results of most commonly used probiotics have shown on pathophysiological symptoms of the liver and many therapies are still under study. Still, there is no treatment for NAFLD. A number of studies about this disease come from animal models and most of the experimental studies have used a single strain of probiotics. A limited data on human studies are available, therefore a large scale of human studies using a combination of specific probiotics are required, which can strongly reduce or even eliminate the prime factors of this disease and can maintain the normal microbiota of the gut. In addition, large-scale, efficient randomized clinical trials (RCTs) might be needed particularly, using advanced tools like omics technologies, to elucidate the exact mechanism(s) of action and achievable therapeutic avenues for NAFLD.

## Figures and Tables

**Table 1:** Impacts of NAFLD on the composition of gut microbiota

Human	Animal	Gut Microbiota Composition in NAFLD					[Ref:]
		Phylum (↑/↓)	Class	Family (↑/↓)	Genus (↑/↓)	Species (↑/↓)	
√		↑*Actinobacteria* ↑*Bacteroidetes* ↑*Firmicutes* ↑*Proteobacteria*			↑*Escherichia*		11
√		↓*Bacteroidetes*				↑*Clostridium coccoides*	37
	√					↓*Akkermansia muciniphila*	38
√		↑*Firmicutes* ↑*Proteobacteria*		↑*Peptostreptococcaceae*	↑*Escherichia* ↑*Lactobacillus* ↑*Streptococcus* ↑*Anaerobacter*		39
√		↑*Bacteroides*			↑*Ruminococcus* ↓*Prevotella*		19
	√	↑*Bacteroides* ↑*Firmicutes*			↑*Atopobium* ↑*Desulfovibrio*	↑*Bacteroides acidifaciens* ↑*Clostridium cocleatum*	40
√		↑*Proteobacteria*		*↑Lachnospiraceae* *↑Enterobacteriaceae*	↑*Fusobacteria* ↓*Prevotella* *↑Blautia* *↑Escherichia* *↑Shigella*		41
√		↑*Actinobacteria* ↓*Bacteroidetes*		↓*Rikenellaceae*	↑*Bradyrhizobium* ↑*Anaerococcus* ↑*Peptoniphilus* ↑*Ruminococcus* ↓*Oscillopira* ↓*Rikenellaceae*	↑*Propioni bacterium acnes*	42
√		↑*Bacteroides*			↑*Lactobacilli* ↓*Ruminococcu* ↓*Bifidobacterium* ↑*Prevotella* ↓*Faecalibacterium*		43
√					↑*Lactobacilli* ↓*Bifidobacteria*	↑*L. mucosae* ↓*B.longum* ↓*B.adolescent* ↓*B.bifidum*	33
√		↑*Firmicutes* ↑*Bacteroidetes*		↑*Proteobacteria*		↑*E. coli*	44
√		↑*Bacteroides*		↑*Enterobacteriaceae* ↑*Ruminococcaceae*	↑ *Streptococcus* ↓*Akkermansia* ↓*Bifidobacterium*		45
√		↓*Firmicutes*		↓*Desulfovibrionaceae* ↓*Leuconostocaceae*	↓*Weissella* ↓*Lactobacilli*		46

**Table 2:** Role of probiotic strains in experimental studies to treat NAFLD

Probiotic strain(n)	Studies/Model	Study Duration	Outcome after treatment/Main Effects	Year-Author [Ref:]
*Lactobacillus reuteri* GMNL-263	High fructose diet (HFD) fed rats	14 weeks	Improve the gut microbiota, antioxidant enzymes, IL-6, TNF-α levels, PPAR-γ, GLUT4 mRNA expression level and overall improving hepatic steatosis	Hsieh—2013 145
*Lactobacillus rhamnosus* GG	Mice: High fructose diet induced NAFLD	8 weeks	Keep mice from NAFLD, improve gut beneficial bacteria, liver enzymes, diminished liver inflammation as well as steatosis	Yvonne—2014 140
*Lactobacillus Rhamnosus* LGG, *Lactobacillus plantarum* WCFS1	Rats: High fat diet induced NAFLD	21 weeks	Ameliorate the intestinal microbiota, gut barrier structures, level of gut endotoxemia, inflammation, lipid metabolism and IR via enhancing the expression levels of CYP7A1 and LDL-R in liver	Lu—2015 147
Probiotic mixture	Mice: HSHF diet induced NAFLD	6 weeks	Alleviate the body mass, blood triglyceride level, total cholesterol level, highest anti-obesity, antioxidant properties	Song—2016 135
Probiotic mixture	Rats: HFD-induced NAFLD	12 weeks	Improve the gut microbiota, delay the development of NAFLD through LPS/TLR4 signaling pathway, improve hepatic fats accumulation and liver enzymes	Xue—2017 139
Probiotic mixture	Rats: High fat diet induced NAFLD	5 weeks	Enhance the gut microbiota composition, liver functions, cholesterol level, oxidative stress and lipid metabolism	Bubnov—2017 146
Probiotic mixture	HFD-induced obese mice	12 weeks	Improve the gut microbiota, up-regulate fatty acid oxidation related genes (PPARα, AOX), in liver and adipocytes, alleviated IL-6 levels	Kim —2017 138
*Lactobacillus plantarum* NA136	HFD/ fructose diet induced NAFLD mice	16 weeks	Ameliorate NAFLD, prevent *de novo* lipogenesis, increase fatty acid oxidation. Reduced weight gain, decreased lipids, mass of tissues fat, serum ALT, AST levels	Zhao—2019 134
*L. acidophilus*, *Bifidobacterium longum*, *Enterococcus faecalis*	C57BL/6J mice: HFD fed mice	4 weeks	Probiotics mitigate the diet-induced obesity, restored the beneficial gut bacteria, reducing serum total cholesterol level, triglycerides and LDL cholesterol	Kong—2019 144
*Lactobacillus plantarum* mixture	Wistar rats: High fat and sucrose diet induced nonalcoholic fatty liver disease	8 weeks	Mitigate the pathogenesis and steatosis in NAFLD through regulating liver function and gut microbiota, downregulated* de novo* lipogenesis associated genes and increased antioxidant enzymes	Park—2020 141
*Bifidobacterium animalis* subsp. *lactis* V9	Wistar rats: high-fat diets induced NAFLD	9 weeks	Regulate the liver function enzymes, ameliorated hepatic steatosis, suppressed inflammatory cytokines, modulate accumulation of the hepatic triglyceride, free fatty acid, increase glycogen level. Decreased serum glucose level, restored the expression of hepatic phosphorylated-AMPK, and reduced the transcription of PPAR-α, SREBP-1c, FAS	Yan—2020 136

LDL: low-density lipoproteins; ALT: alanine transaminase; LPS: lipopolysaccharide; AST: aspartate aminotransferase; TLR4: toll-like receptor 4; PPARα: peroxisome proliferator-activated receptor-α; GLUT4: insulin-regulated glucose transporter; AOX: alternative oxidase; CYP7A1: cholesterol 7α-hydroxylase; ZO: zonula occludens; AMPK: adenosine monophosphate activated protein kinase; SREBP-1c: sterol regulatory element-binding transcription factor 1; FAS: fatty acid synthase.

**Table 3:** Role of probiotic strains in clinical studies to treat NAFLD patients

Probiotic strain(n)	Studies/Model/ patients	Study Duration	Outcome after treatment/Main Effects	Year-Author [Ref:]
*Lactobacillus rhamanus*	20 Obese children	8 weeks	Reduce the body weight, alleviate ALT, AST and tumor necrosis factor-alpha levels	Vajro—2011 157
*Bifidobacterium longum+ Fructo-oligosaccharides (FOS)*	Randomized, double blind and placebo controlled trial, 66 NAFLD (biopsy proven) patients	24 weeks	Improve TNF-α, C-reactive protein, AST, endotoxin levels, HOMA-IR, liver steatosis and NASH	Malaguarnera—2012 150
*Lactobacillus acidophilus (La5), Bifidobacterium lactis (Bb12)*	Double-blind, randomized and controlled trial, 72 patients with NAFLD	8 weeks	Alleviate AST, alanine aminotransferase, serum triglycerides (TC), total cholesterol and LDL cholesterol	Nabavi—2014 158
*Lactobacillus Acidophilus, Bifidobacterium bifidum, Bifidobacterium lactis* and *Lactobacillus rhamanus*	Randomized, triple blind trial, 64 children with obesity-related liver syndromes(NAFLD)	12 weeks	Enhance liver function enzymes level, reduced lipopolysaccharide levels, triglycerides	Famouri—2017 149
Probiotic mixture* (Bifidobacterium Lactobacillus & Enterococcus*,* B. subtilis* and *Enterococcus)*	200 NAFLD patients	1 month	Ameliorate the gut microecological structure in NAFLD patients, decreased serum ALT, AST levels, inhibiting TNF-α level and enhancing adiponectin level. Improved fatty liver, blood lipids and blood glucose level, lipid metabolism and protect liver injury	Wang—2018 57
*Bifidobacterium animalis*	34 NAFLD patients	24 weeks	Alleviate hepatic steatosis, serum ALT, aspartate aminotransferase level	Bakhshimoghaddam—2018 156
Probiotic mixture	68 obese NAFLD patients	12 weeks	Reduce body weight, total body fat, decreased triglyceride, ALT, AST levels	Ahn—2019 159

TNF-α: tumor necrosis factor alpha; HOMA-IR: homeostatic model assessment of insulin resistance; IL-6: interleukin 6; IL-10: interleukin 10; LDH: lactate dehydrogenase; CRP: c-reactive protein; IR: insulin resistance; HDL-cholesterol: high-density lipoproteins-cholesterol; LDL-cholesterol: low-density lipoprotein cholesterol; TG: triglycerides.

## Abbreviations

HFHFD: high fat and high fructose diet
SIBO: small intestine bacterial overgrowth
BT: bacterial translocation
TJ: tight junction
FFA: free fatty acid
IR: insulin resistance
ALD: alcoholic liver disease
NAFLD: nonalcoholic fatty liver disease
ASH: alcoholic steatohepatitis
NASH: nonalcoholic steatohepatitis
SCFA: short chain fatty acid
VLDL: very low-density-lipoproteins
LPL: lipoprotein lipase
LPS: lipopolysaccharide
Th: T helper cells
Treg: regulatory T cells
Ig: immunoglobulin
IL: interleukins
TNF: tumor necrosis factor
TGF: transforming growth factor
IFN: interferon
DC: dendritic cell
SCFA: short chain fatty acid
M: macrophage
NF-Κb: nuclear factor-κB
MAPK: mitogen-activated protein kinase
ROS: reactive oxygen species
